# MicroRNAs and Epigenetics Strategies to Reverse Breast Cancer

**DOI:** 10.3390/cells8101214

**Published:** 2019-10-08

**Authors:** Mohammad Mijanur Rahman, Andrew C. Brane, Trygve O. Tollefsbol

**Affiliations:** 1 Department of Biology, University of Alabama at Birmingham, 1300 University Boulevard, Birmingham, AL 35294, USA; mijanur@uab.edu (M.M.R.); brane@uab.edu (A.C.B.); 2 Comprehensive Center for Healthy Aging, University of Alabama Birmingham, 1530 3rd Avenue South, Birmingham, AL 35294, USA; 3 Comprehensive Cancer Center, University of Alabama Birmingham, 1802 6th Avenue South, Birmingham, AL 35294, USA; 4 Nutrition Obesity Research Center, University of Alabama Birmingham, 1675 University Boulevard, Birmingham, AL 35294, USA; 5 Comprehensive Diabetes Center, University of Alabama Birmingham, 1825 University Boulevard, Birmingham, AL 35294, USA

**Keywords:** microRNAs, epigenetics diet, epigenetic editing, hTERT, estrogen receptor

## Abstract

Breast cancer is a sporadic disease with genetic and epigenetic components. Genomic instability in breast cancer leads to mutations, copy number variations, and genetic rearrangements, while epigenetic remodeling involves alteration by DNA methylation, histone modification and microRNAs (miRNAs) of gene expression profiles. The accrued scientific findings strongly suggest epigenetic dysregulation in breast cancer pathogenesis though genomic instability is central to breast cancer hallmarks. Being reversible and plastic, epigenetic processes appear more amenable toward therapeutic intervention than the more unidirectional genetic alterations. In this review, we discuss the epigenetic reprogramming associated with breast cancer such as shuffling of DNA methylation, histone acetylation, histone methylation, and miRNAs expression profiles. As part of this, we illustrate how epigenetic instability orchestrates the attainment of cancer hallmarks which stimulate the neoplastic transformation-tumorigenesis-malignancy cascades. As reversibility of epigenetic controls is a promising feature to optimize for devising novel therapeutic approaches, we also focus on the strategies for restoring the epistate that favor improved disease outcome and therapeutic intervention.

## 1. Introduction

Breast cancer is a common cancer in women worldwide and was the highest (11.6%) newly diagnosed cancer type last year second only to lung cancer [1]. The trend of survival and mortality rates of breast cancer show geographical variation. Whereas breast cancer mortality is decreasing in North American and European countries, its mortality rate has been increasing in many Asian, African, and Latin American countries since the 1990s [2]. In the United States, the incidence rate of breast cancer shows a nearly steady trend, whereas the mortality started declining gradually from 1990 [3]. This decrease in mortality rate may be attributed to earlier cancer diagnosis and improved therapeutic intervention [2].

Principally, breast cancer therapy aims for tumor regression from the breast and/or halting the spread of the cancer to distal anatomical locations through metastasis. The available therapeutic strategies against breast cancer are either systemic or local in nature. Systemic therapy often targets both nonmetastatic and metastatic breast cancer through endocrine therapy and chemotherapy, whereas nonmetastatic breast cancer is frequently targeted by local therapy such as surgery and radiation. In many breast cancer cases, systemic and local therapies are used in combination [4]. The choice of the treatment strategies largely depends on the molecular subtyping based on the expression status of estrogen receptors (e.g., ERα and ERβ) and human epidermal growth factor receptor 2 (HER2) as well as breast cancer stage [5].

Although understanding of cancer biology has improved during the last few decades, treating breast cancer remains challenging due to the disease heterogeneity, therapeutic target assortment, therapeutic resistance, residual-disease, and breast cancer recurrence even after targeted therapy [6]. Interestingly, the genetic background fails to explain the molecular anomaly of breast cancer entirely due to the sporadicity often associated with breast cancer. The genomic causes responsible for breast cancer are acquired more often than inherited [7]. To date, epigenetic processes like DNA methylation, histone modification, and microRNAs (miRNAs) have been reported to be involved with every aspect of breast cancer pathophysiology, diagnosis, and treatment [8,9,10]. Moreover, several basic and interventional attempts of targeting breast cancer with epigenetic drugs have recently proven successful [11,12,13,14,15]. Indeed, an epigenetic view of breast cancer is crucial to explain the molecular basis of breast cancer, improve therapeutic strategies, and to develop new therapeutic tools against breast cancer. All these factors contribute to moving beyond the genetic framework of breast cancer toward the epigenetic concept of breast cancer.

In this review, we will first go through an account of the epigenetic changes frequently associated with breast cancer, including DNA methylation, histone modification, and miRNAs with updated evidence. In order to illustrate the epigenetic profiles of breast cancer, we have considered epigenetic regulation in two different contexts (1) dynamic epigenetic regulation as exhibited by estrogen receptor (ER) expression in breast cancer and (2) noncannonical epigenetic regulation as shown by human telomerase reverse transcriptase (hTERT) expression during breast cancer. We will also focus on how breast cancer-associated epigenetic changes sustain tumorigenesis and metastasis as well as cancer hallmarks. Finally, we will cover epigenetic-based strategies against breast cancer such as the employment of epigenetic drugs, epigenetic diets, epigenome editing tools, and miRNA-based therapy.

## 2. Breast Cancer Epigenetics

Epigenetic reprogramming is a common feature of breast cancer irrespective of the inaugural genetic causes. While initial genetic mutation leading to multistep breast tumorigenesis is not fixed to a certain oncogene or tumor suppressor gene, epigenetic aberrations always follow the genetic destabilization [16]. Alterations of DNA methylation and histone modification marks, as well as miRNAs expression, are the most comprehensively studied epigenetic changes in the breast cancer context.

### 2.1. DNA Methylation

DNA methylation denotes covalent and reversible addition of a methyl group from S-adenosyl methionine (SAM) to the fifth carbon position of the cytosine ring on genomic CpG dinucleotides. A group of enzymes known as DNA methyltransferases (DNMTs) function as a writer to inscribe the methylation marks on the genomic DNA, whereas ten-eleven translocation (TET) family enzymes (i.e., TET1, TET2, and TET3) can erase the existing methylation marks through their methylcytosine dioxygenase activity [17]. DNMT1, DNMT3A, and DNMT3B possess DNA methylation ability among the members of the DNMT family. DNMT1 methylates only hemimethylated DNA ensued from DNA replication to restore the template DNA methylation patterns and is hence known as the maintenance DNA methyltransferase. DNMT3A and DNMT3B are referred to as the de novo DNA methyltransferases because they catalyze the methylation of genomic DNA afresh to establish new DNA methylation patterns during embryogenesis [18]. These DNMTs work in harmony to maintain a healthy methylation pattern for sustaining a balanced transcriptional control over the genome. One of the pioneering studies from our lab suggested the upregulation of DNMTs (DNMT1, DNMT3A and DNMT3B) as a mechanism underlying global methylation profile change in neoplastically transforming cells [19]. Indeed, methylome reshaping during tumorigenesis is considered an enabler for neoplastic transformation. Generally, the methylome experiences a global hypomethylation in parallel with region-specific hypermethylation during the early stages of tumorigenesis [20,21].

Aberrant DNA methylation patterns in breast cancer were an obvious finding from the pre-genome-wide association studies (GWAS) era. The advent of whole-genome approaches about a decade ago enhanced the linkage between DNA methylation patterns and breast cancer pathogenesis. One of the earliest studies done by Han et al., identified 345 differentially methylated genes in 40 different breast cancer cell lines upon genome-wide methylation profiling [22]. However, methylome profile shifting is more extensive in breast cancer tissues compared to breast cancer cell lines. For example, Wang et al., (2014) reported 2753 hypomethylated genes and 1795 hypermethylated genes in breast cancer after examining fresh patient-derived tissue samples [23]. In a recent study, Holm et al., (2016) investigated the link between methylome pattern variation and breast cancer heterogeneity. They reported differential methylation of about 18,700 genes [24]. Methylome heterogeneity appraisal suggested a linear correlation between the target CpG coverage and reported differentially methylated regions (DMRs) from the study [24,25,26]. The location and regulatory consequence of the breast cancer-associated DNA methylome divergence follow canonical consequence. Similar to many other cancer scenarios, hypermethylation predominated in promoter upstream regions, whereas hypomethylation was primarily located within gene bodies in the case of breast cancer [27]. The promoter CpG methylation typically correlates negatively with gene expression. However, when it localizes within the gene body, CpG methylation can lead to transcriptional activation [27,28,29].

The influence of aberrant DNA methylation in breast cancer is extensive throughout the trajectory of neoplastic transformation to metastasis. During tumorigenesis, early DNA methylation shuffling affects genes involved in cell differentiation, DNA binding, homeobox proteins and transcription signaling to stimulate the tumor-promoting properties of breast cancer stem cells [26,30,31]. About 800 low activity promoters are hypermethylated including 48 gene sets associated with cancer, polycomb regulation and transcription factors besides 280 high activity promoters hypomethylation in an early basal-like breast carcinogenesis model (i.e., vHMEC) [31]. A host of genes involved with chromatin remodeling, transcriptional control, DNA repair, cell-cycle control, apoptosis and metabolism (e.g., gluconeogenesis) undergo a change in methylation during the growth of neoplastic cells [8,25,32,33,34]. As the transformed cell proceeds toward malignancy, the DNA methylation profile alters cell adhesion, tissue invasion, and metastasis pathway genes [8,33]. DNA methylation divergence even holds influence over the tumor microenvironment. For example, a study by the Mathot team showed DNA methylation pattern change of the stromal-dependent genes to modify the responsiveness of breast cancer cells themselves toward the microenvironment [35]. This finding expands the potential influence of DNA methylation beyond the molecular framework of the tumorigenesis-metastasis axis.

### 2.2. Histone Modification

Histones are highly conserved DNA-packaging proteins that form an octameric cylindrical scaffold to facilitate the wrapping of ~147-bp DNA stretched around the cylindrical spool. The octamer core consists of two sets of core histone protein H2A, H2B, H3, and H4. Core histone proteins assemble in a manner to form a central globular histone fold comprising ~75% of the core histone protein mass. The rest of the histones mass forms largely amorphous, flexible histone tails [36]. The tails (H3/H4 N-terminal tail and H2A/H2B C-terminal tail), as well as the histone, fold harbor sites for reversible and covalent post-translational modifications like acetylation, methylation, ubiquitination, phosphorylation, sumoylation, poly-ADP-ribosylation, neddylation and glycosylation [37,38]. The histone modifications during tumorigenesis and metastasis have been studied widely in the context of cancer for developing biomarker, prognostic tools, and therapeutics [39,40,41].

Histone acetylation and methylation are the most studied histone modifications in breast cancer [9,10,42,43]. An acetyl group from acetyl-CoA is covalently added to the amino group of lysine residues on histone tails during histone acetylation. The exchange of the amino group with the acetyl group neutralizes the net basic charge of the native unmodified lysine residue. As a result, the electrostatic interaction between negatively charged DNA and positively charged histones weakens which, in turn, leads to chromatin decondensation and a more accessible transcriptionally active chromatin structure [44]. Removal of an acetyl group usually reverses the chromatin loosening effect of histone acetylation and causes transcriptional repression. The histone acetyltransferases (HATs) catalyze the acetylation of lysine residues on the histones, whereas histone deacetylases (HDACs) remove the acetylation signature from the histones. On the other hand, histone methylation refers to the covalent addition of a methyl group to the lysine and arginine residues on histone tails. The replacement of an amino group with a methyl group, being hydrophobic in nature, interferes with the basicity/hydrophobicity of the subject residue. Ultimately, the basicity/hydrophobicity shift affects the interaction of DNA with protein molecules such as transcription factors [45]. Similar to histone acetylation, the histone methyltransferases (HMTs) catalyze the methylation, whereas histone demethylases (HDMs) erase the methyl group from the methylated histone lysine/arginine residues [41].

Attributes of histone modifications on gene expression are diverse. The readout from the acetylation modification is straightforward where acetylation denotes a transcriptionally active euchromatin state and vice versa. For example, the acetylation of lysine K5, K8, K9, K12, K14, K18 residues of histone H3 and K16 residue of histone H4 represents open chromatin status and gene activation [14]. The functional output of histone methylation depends on the histone type and residue incurring the modification. For instance, H2BK4me, H3K4me3, H3K20me, H3K36me3, and H3K79me3 modifications are associated with active chromatin configurations, whereas H2BK5me3, H3K9me3, H3K27me3, and H4K20me3 are associated with gene repression [37]. In many cases, the transcriptional readout from the histone modification is complex where the gene expression relies on modification of multiple amino acid residues within a cassette rather than a single residue and modification nature [46].

The histone modification profile shuffling during breast cancer predicts the disease prognosis. The profiling of the global changes in histone acetylation (i.e., H3K9ac, H3K18ac, H4K12ac, and H4K16ac) and methylation (H4R3me2, H3K4me2, and H4K20me3) markers in normal and primary invasive breast carcinoma suggested a correlation between the histone modification profile alteration and tumor prognosis. The depletion of H3 and H4 acetylation marks featured a worse prognosis, while the accumulation of acetylation marks predicted better tumor prognosis [47]. Besides conventional molecular markers, the histone modification profile is an important subtyping marker for breast cancer. The immunohistochemical staining of tumor tissue microarray revealed global H3K27me3 enrichment in luminal A subtype but H3K27me3 depletion in HER2-type and basal-like breast cancer [48]. A genome-wide distinct pattern of H3K4me3 and H3K27me3 marks was apparent in the breast cancer cell lines representing luminal, HER2-enriched, and basal subtypes of breast cancer [49]. Indeed, analysis of histone post-translational modification introduced the idea of “clustering” as an extended dimension of breast cancer subtyping. Depending on the genome-wide K27me3/K9me3 ± K14ac level, the breast cancer basal subtypes were grouped into three separate clusters [50].

The overall histone modification landscape of breast cancer exhibits an assorted profile. A comprehensive study by Noberini et al., profiled three H3 lysine acetylation (K4, K14, K79), fourteen H3 lysine methylation (K4me2, K4me1, K9me1, K9me2, K9me3, K18me1, K27me1, K27me2, K27me3, K36me1, K36me2, K36me3, K79me1, K79me2) and fourteen combinatorial lysine modifications (e.g., H3K9Ac/K14Ac, H3K9me2/K14Ac, H3K27me2/K36me2 etc.) using patient-derived breast cancer specimen tissue, primary breast cancer cells, and available breast cancer cell lines. In general, the histone modification profile of the frozen tissue was distinct from those of the primary cell lines and established breast cancer cell lines. Multivalent lysine methylation patterns were distinct among the studied sample cohorts though the other histone modification pattern of the primary cell lines and established breast cancer cell lines were similar to each other. Nonetheless, several changes in histone modification trended to show some degree of exclusiveness toward the cell line type. For example, K36me1 or K9me3/K14Ac deposition was more frequent in breast cancer cell lines, while primary cells were inclined to lose the K14Ac mark [51].

Genome-wide association studies of monovalent/multivalent histone modification have contributed to understanding the impact of histone modifications on breast cancer initiation and progression. An early study by Zhao et al., (2016) explained the impact of H3K9 posttranslational modification on neoplastic transformation during breast cancer using a transformation model that stably expresses the Large T antigen, TERT, and RAS (V12). The tumorigenic transformation was marked by the reduction of H3K9me2/me3 simultaneously with H3K9ac accumulation. As an outcome, the authors suggested a transcriptional reprogramming of about 650 genes that directed the normal cellular processes toward neoplastic transformation [52]. Intriguingly, the imbalance of histone modifications has been implicated with the tumorigenesis, cancer progression, and metastasis of breast cancer. The work of Karsli-Ceppioglu and colleagues provided an overview of the influence of histone modifications on breast cancer signaling where the group studied genes that underwent H3K9ac/H3K27me3 dysregulation in breast tumor tissues. The H3K9ac imbalance was associated with the genes involved with cell proliferation, apoptosis regulation, cell-cell signaling, cell migration, and metabolic process, while cell cycle-associated genes were enriched with H3K27me3 [53]. Moreover, the chemotherapy resistance could be attributed to histone modification alteration, as the lack of H3K27me3 was commonly featured by drug resistance and resistant-like cells [54].

### 2.3. MicroRNAs (miRNAs)

#### 2.3.1. MicroRNAs and Cancer

MicroRNAs (miRNAs) are 17–25 nucleotides long, single-stranded, non-coding, regulatory RNAs that regulate gene expression at the post-transcriptional level. A recent high-throughput analysis estimated about 2300 true mature miRNAs in the human genome [55]. The genomic distribution of miRNAs suggests that the miRNAs genes are intragenic, intronic, or exonic [56]. The biogenesis of the miRNA largely depends on the genomic organization of miRNAs. Except for the mirtrons, a type of intronic miRNAs generated from the pre-miRNA splicing by the spliceosome, most of the miRNAs are transcribed by RNA polymerase II to primary miRNA (pri-miRNA). The pri-miRNAs are processed by Dorsa microprocessor complex to ~70 nucleotide-long hairpin structures known as precursor miRNA (pre-miRNA). The pre-miRNAs are exported to the cytoplasm and cleaved to ~22 nucleotide duplex mature miRNA by Dicer–TRBP–Ago2 complex. Once matured, both of the miR strands could direct downstream gene regulation. Either the 5′ strand (miR-5p) or 3′ strand (miR-3p) is loaded to the RNA-induced silencing complex (RISC) [57].

The miRNAs recognize their targets by the seed region that spans from the 2 to 8 position at the 5′ end of miRNAs. This seed region can identify the seed sequence on target mRNA which usually locates at the 3′ untranslated region (UTR) of the target mRNA. The cytoplasmic effect of the miRNAs is executed by the RISC either by RNA interference or by translational repression. The output of the RISC activation largely depends on the sequence complementarity between miRNA and target mRNA. A perfectly/nearly perfectly miRNAs to target mRNA match results in target mRNA digestion by RISC. Otherwise, a poor match between miRNAs and target mRNA leads to the destabilization of target mRNAs. The target mRNAs destabilization roughly represses 6–25% of global protein expression [58].

Among the earliest research of miRNA on cancer was in 2002 by the Calin group who reported the down-regulation of miRNA-15 and miRNA-16 in chronic lymphocytic leukemia [59]. From the start, a surge of empirical studies commenced to investigate the role of miRNAs in cancer. Numerous studies have reported miRNAs dysregulation in cancerous cell proliferation and growth, cell death inhibition, immune invasion, metastasis, and neoangiogenesis [60]. There is recent evidence suggesting that miRNAs regulate the fate of cancer stem cells (CSCs). A panel of stem cell miRNAs target and balance the expression of genes, including Octamer-binding transcription factor 4 (*OCT 4*), NANOG, SRY-Homeobox 2 (*SOX2*) and *NOTCH*. For exemple, *SOX2* is targeted by miR-200 family members, including miR-200a, miR-200b, miR-200c, miR-141, and miR-429. MiR-34a expression correlates negatively with CSC differentiation, whereas miR-34a can target Notch1 directly. The hedgehog signaling pathway, crucial for controlling the CSC behavior, could be targeted by miR-125b, miR-324-5p, and miR-326. Since these genes are essential for pluripotency and associated stem cell processes, dysregulation of stem cell miRNAs causes enhanced self-renewal and reduced apoptosis of CSCs [61].

**Figure 1:**
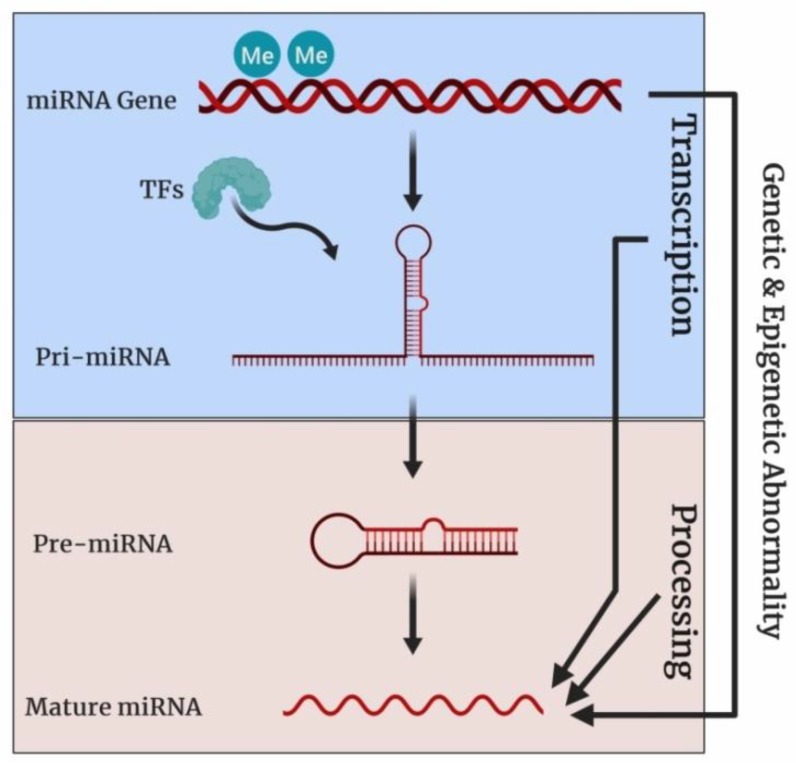
Molecular mechanism of miRNA dysregulation during cancer. Here, “Me” indicates CpG methylation, “TFs” indicates transcription factors. The expression of miRNA genes is regulated at three different levels: Genetic and/or epigenetic, transcriptional, and bioprocessing level. At the genetic level, the copy number of miRNA genes may change due to the amplification/deletion of miRNA genes. CpG hypermethylation, a common epigenetic change during cancer, may control the miRNA genes expression aberrantly. At the transcriptional level, cancer-associated shuffling of transcription factors may alter miRNA expression profiles. Finally, miRNA expression is influenced by any dysregulation of components of the miRNA biosynthesis cascade (e.g., Drosha, Dicer, DGCR8, Argonaute proteins and exprotin 5).

The molecular mechanism underlying aberrant miRNA expression during cancer has been summarized in Figure 1. The miRNA dysregulation during cancer may result from alteration of genetic and/or epigenetic regulation of the miRNA genes, disruption of miRNA transcriptional control, and aberrant miRNA biogenesis [60]. Cancer-associated genomic instability often leads to miRNA copy number variation due to the amplification or deletion of miRNA genes. The hypermethylation of CpG islands near the miRNA genes induces epigenetic silencing and consequential transcriptional repression [62]. Moreover, epigenetic drugs have been reported to alter the expression profile of miRNAs. For example, combinatorial treatment with HDAC inhibitor suberoylanilide hydroxamic acid (SAHA) and DNMT inhibitor epigallocatechin gallate (EGCG) down-regulated the oncogenic miRNA-221/222 expression in triple-negative breast cancer cell lines (i.e., MDA-MB-157 and HCC1806) [63] in different breast cancer cell lines. Another study has reported a reduction in oncogenic miRNA-21 expression in colon cancer cells line RKO from treatment with SAHA [64]. In normal cells, miRNA expression is strictly regulated by a set of transcription factors like p53, c-Myc, E2F, Twist, STAT3, etc. [62]. During malignancy, an anomaly in the expression and functionality of these transcription factors causes the alteration in the expression of the miRNAs involved in various cellular processes including cell growth, proliferation, apoptosis, metastasis, and neoangiogenesis. Another level of regulation of miRNA expression occurs during miRNA biosynthesis. The enzymes and regulatory proteins (e.g., Drosha, Dicer, DGCR8, Argonaute proteins, and exprotin 5) that participate in miRNA biogenesis may undergo mutation or abnormal expression during tumorigenesis and malignancy [60]. Dysregulation of the miRNA biogenesis machinery, usually, leads to an abnormal miRNA expression profile.

Besides a canonical role in gene silencing, miRNAs are able to induce the expression of target genes through a process known as RNA activation (RNAa) [65]. Gene activating miRNAs are better known as small activating RNAs (saRNAs). Though saRNAs exploit components of RNA interference (RNAi) pathway such as Dicer and Argonaute (Ago) family members, the exact mechanism of RNAa-mediated gene activation is unclear. Mechanistically, saRNAs binding to a promoter facilitates RNA Polymerase II (RNAPII) assembly on the target promoters. The initial assembly of RNAPII is followed by heterogeneous nuclear ribonucleoproteins (hnRNPs), specific coactivators and chromatin remodeling factor recruitment and eventual gene activation [66]. In addition to promoters, other gene regulatory units such as enhancers and suppressors could be targeted by saRNAs [67,68]. Recently, Xiao et al., showed that saRNAs (e.g., miRNA-24-1) triggered enhancer activation through RNA polymerase II, p300/CBP and enhancer RNAs enrichment [67]. A number of in vitro studies have shed light on the role of saRNAs in cancer. Depending on the downstream target, saRNAs are either tumor-suppressive or oncogenic in nature. For instance, tumor suppressor p16 expression was increased by miR-877-3p with concomitant reduction of proliferation and tumorigenicity in bladder cancer cell lines (5637, UM-UC-3, and T24) [69]. MiR-6734 induced the expression of tumor suppressor p21 along with enhanced cell cycle arrest and apoptosis in HCT-116 colon cancer cells [70]. On the contrary, miRNA-551b-3p upregulates oncogene STAT3 expression and promoted the proliferation and survival of ovarian cancer cells (i.e., IGROV1, IOSE80, and HEYA8) [71]. Recently, a comprehensive study was conducted by Tan et al., to demonstrate the importance of miRNA-associated gene activation in a multicancer scenario. The authors intended to reveal the positive association between miRNAs and gene expressions using a large panel of patient samples of 31 major human cancers types. They reported a cohort of about 340 miRNAs capable of targeting the activation of about 3074 genes. The target genes included the gene sets involved in biological processes crucial for tumorigenesis and metastasis such as cell growth and development, metabolism and cellular immunity. Largely, the reported miRNAs (~340) impacted every hallmark of cancer [68].

#### 2.3.2. MicroRNAs and Breast Cancer

MiRNA dysregulation is associated with the progression through every stage of breast cancer, beginning with tumorigenesis progressing to metastasis through proliferation and progression. In line with many other cancer types, the study of miRNAs in a breast cancer context increased exponentially after 2005. Presently, a number of reviews on the role of miRNAs in breast cancer have updated the investigational status. Due to new techniques and promising therapeutic potential of miRNA, many new studies are conducted each year investigating the connection of miRNA with breast cancer. These new studies are summarized below in Table 1. The miRNAs profile anomaly was vast enough to affect the cellular signaling pathways of proliferation, growth, apoptosis, epithelial–mesenchymal transition (EMT), metastasis, and angiogenesis. The tumor-suppressive miRNAs inhibited the cancerous cellular process, whereas other miRNAs were oncogenic in nature. Most importantly, the effect of a panel of miRNAs is limited to a particular cell signaling cascade, while others target breast cancer globally.

Beyond the molecular function, miRNAs provide important information for breast cancer subtyping, diagnosis, prognosis, and treatment monitoring. The first attempt to subtype breast tumors according to miRNA expression was taken by Blenkiron et al. (2007). They detected assorted expression of 133 miRNAs in normal and cancerous breast tissue after analyzing the expression profiling of 309 miRNAs in normal and tumorous primary breast tissue. More strikingly, differential expression profiles of miRNAs allowed categorizing breast cancer according to molecular breast tumor subtypes: luminal A, luminal B, basal-like, HER2^+^ and normal-like (Table 2) [159]. Since this report, a panel of miRNAs has been developed as breast cancer subgrouping biomarkers and investigations are continuing to discover and optimize miRNA panel that can be utilized as subtyping biomarkers more concisely [8].

Another set of miRNAs may inform breast cancer progression, prognosis, and recurrence in patients [160]. For example, expressional analysis of miRNA-10b, miRNA-34a, miRNA-373, miRNA-21, and miRNA-155 provides clinical information about tumor stage and/or metastasis [160,161]. The analysis of miRNA-18b, miRNA-103, miRNA-107, and miRNA-652 expressions in serum samples predicts tumor relapse risk and overall survival in triple-negative breast cancer patients as an independent prognostic classifier [160]. The disease-free survival of breast cancer patients was associated with high levels of miRNA-93, miRNA-195, and miRNA-20b expression [162]. Furthermore, recent studies demonstrate that miRNAs have the potential to predict the response of breast cancer to systemic treatments. For instance, the expression status of miRNA-342-3p and miRNA-187-3p was linked to systemic treatment success. High-level expression of miRNA-342-3p and miRNA-187-3p support progression-free survival and overall survival [163]**.**

### 2.4. Estrogen Receptors

Numerous studies have established estrogens as key hormones involved in both normal breast development and breast tumor formation. The physiological functions of the estrogens are executed by intracellular estrogen receptors (ERs). Two different isoforms of ERs have been characterized: *ESR1* encodes estrogen receptors α (ERα) and *ESR2* encodes estrogen receptors β (ERβ). The estrogen receptors signaling could follow one of two cascades: genomic and non-genomic. During the genomic signaling cascade, ERs dimerize as a result of conformational changes from estrogens binding before they bind to estrogen receptor elements (EREs) of target genes [164] with consequential gene regulation. The nongenomic signaling pathway involves second messengers and effector proteins (and kinases) activation [165]. Though nongenomic signaling from ERs is capable of stimulating endothelial cell proliferation, this mode of signaling has been shown uncoupled with a breast cancer-associated function of ERs [166,167].

**Figure 2:**
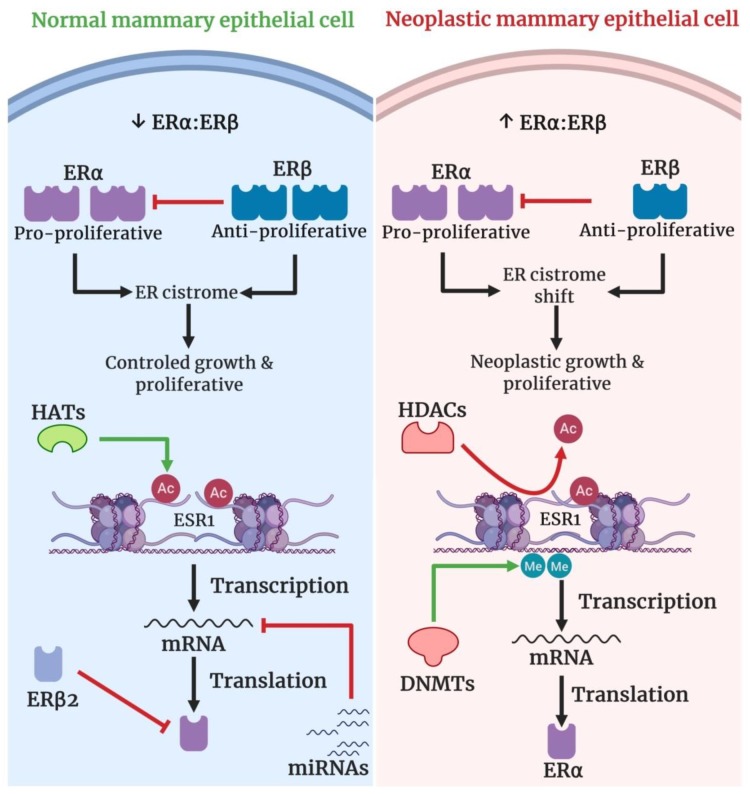
Proposed model for coordinated epigenetics regulation of ER isotype (ERα and ERβ) expression dynamics in breast cancer. A reciprocal expression profile of ERα verses ERβ on ductal and lobular epithelial cells establishes initial low ERα: ERβ expression. The resultant ERα: ERβ sustains an ER cistrome supportive of normal cellular growth and proliferation. The original ERα: ERβ is set by several layers of epigenetic control; *ERα* promoter demethylation, *ERα* promoter acetylation, miRNA homeostasis, and ERβ2 induced proteasome degradation of ERα [175]. Neoplastic transformation moves the ERα: ERβ from low to high score which, in turn, results in ER cistrome shift favorable for neoplastic grow and proliferation. *ERα* promoter methylation, loss of ERβ2 assisted ERα level control, *ERα* promoter deacetylation and miRNA expression profile shift together lead to ERα down-regulation as the cells undergo neoplastic transformation. Though ERβ down-regulation contributes profoundly to the ERα:ERβ score, the exact epigenetic regulations of ERβ expression decrease are yet to be studied. However, the cancer cells acquire more aggressive cancerous phenotypes like estrogen-independent growth as the tumor proceeds through a malignant transformation pathway.

The ERs are used as both bimolecular prognostic tools and endocrine therapy targets. The oncogenic role of ERα induces the neoplastic growth and metabolic reprogramming during breast tumorigenesis [168]. Being tumor suppressive in nature, ERβ activity prevents growth, migration, and invasion of breast cancer cells [169]. About 75% of primary breast cancer cases are diagnosed as ER-positive [170]. In general, high ERα and ERβ expression correlates positively with better clinical outcome and disease recovery. On the other hand, down-regulation of ERα leads to poor disease prognosis, greater malignancy, and low responsiveness to endocrine therapy [171]. The dynamics of ER isoform expression in breast cancer is shown in Figure 2. ERα expression is restricted to ductal and lobular epithelial cells with ERα expression in ~10% of epithelial cells in normal breast tissue. Conversely, ERβ is expressed in ~70% of ductal and lobular epithelial cells besides stromal cells [172,173]. This reciprocal expression of ERs ultimately leads to a low ERα: ERβ expression. Tumorigenesis shifts the overall ERα: ERβ expression from low to high score [173]. This shifting redirects the estrogen signaling from a normal to a tumor-specific version of estrogen signaling. Recently Chi et al., have characterized the estrogen-responsive transcriptome and ER cistrome in normal ER-positive (ER^+^) mammary epithelial cells. They showed that neoplastic transformation leads to transcriptional profiling shuffling upon estrogen stimulation. Interestingly, the transcriptional profile shift was primarily contributed by an active ER cistrome [174]. As the tumor progresses, cancer assumes a more aggressive phenotype like estrogen-independent cancer growth through the loss of ER expression.

The loss of ER expression complicates the selection of treatment strategy against breast cancer. A number of molecular mechanisms have been reported on ER down-regulation and loss in breast cancer: ER gene mutation, ER gene heterozygosity loss, altered transcriptional regulation of *ER* promoter, *ER* mRNA destabilization, *ER* mRNA alternative splicing, ER proteasome degradation, hyperactive GFR/MAPK signaling and epigenetic regulation. The ER expression loss is more epigenetic than genetic in nature [175]. So far, reported epigenetic regulations associated with ER expression loss are (1) *ER* promoter hypermethylation, (2) histone deacetylation, and (3) miRNAs [174,176]. The hypermethylation of the *ER* gene promoter causes ER expression loss, whereas the inhibition of ER gene promoter CpG methylation reactivates the ER expression [174,177,178]. ERα reexpression was induced in ERα-negative MDA-MB-231 cells using DNA methyltransferase (DNMT) inhibitor EGCG [179]. Another layer of ER expression is regulated by histone deacetylation where histone acetylation or inhibition of histone deacetylation upregulates ER expression. For instance, treatment with trichostatin A (TSA), a histone deacetylase (HDAC) inhibitor, reactivated ERα expression in ERα-negative breast cancer cells through down-regulation of HDAC activity and consequential enrichment of the activating H3K9Ac mark on the *ERα* promoter [180]. More recently, we were able to reactivate ERα expression in ERα-negative breast cancer cells by combined treatment with the HDAC inhibitor SAHA and DNMT inhibitor EGCG [64].

miRNAs have been reported to control ER expression in breast cancer via either direct or indirect mechanisms. For instance, miRNA-142-3p binds directly to the 3′ UTR of *ESR1* messenger RNA (mRNA) to down-regulate ER expression in ER-positive breast cancer [91]. Likewise, miRNA-335-5p, miRNA-21 and miRNA-192-5p act directly to down-regulate ER expression [181,182,183]. Though not reported extensively, some miRNAs indirectly control ER expression. For example, miRNA-148a controls the ER expression targeting DNMT1 indirectly. Mechanistically, miRNA-148a reduces DNMT1 expression which, in turn, upregulates ER expression in MCF-7 cells [184].

The oncogenic miRNAs induce ER overexpression and the impact of oncogenic miRNAs on estrogen receptor expression largely depends on the disease context. During the early stages of breast cancer, oncogenic miRNAs-induced ER overexpression usually aggravates cancer progression. In advanced stages of breast cancer, often marked by reduction or loss of ER expression, oncogenic miRNAs induce ER re-expression. The restoration of ER expression during advanced breast cancer improves the sensitivity of breast cancer toward systematic therapy. For example, miRNA-27a induces the expression of ERα. When transfected with miRNA-27a mimics, luminal A breast cancer cells (MCF-7 and T47D cells) exhibited improved sensitivity towards ER modulators tamoxifen, endoxifen and toremifene [185]. Conversely, Luengo et al., reported a poorer survival of breast cancer patients that received neoadjuvant chemotherapy with miRNA-18a expression in residual tumors. Indeed, miRNA-18a expression lowers ER expression and decreases tamoxifen sensitivity [186].

To date, the number of reported miRNAs that can target ER genes (*ESR1 and ESR2*) is limited. Considering the therapeutic value and clinical application, there is interest in adding new ER-targeting miRNA members to the list. While experimental studies are exploring such miRNAs, a number of bioinformatics tools are available to predict the miRNAs capable of targeting ER. Being interested in new ER-targeting miRNAs, Table 3 summarizes a list of predicted miRNAs against *ESR 1* and *ESR 2* using bioinformatics tools [187,188]. Interestingly, a number of miRNAs against *ESR 1 and ESR 2* may target other mRNAs. For example, hsa-let-7a-5p could target four other genes (e.g., *BZW1*, *COL1A2*, *DUSP1*, and *HMGA2*) with high confidence (Integrated Score >0.95) being a highly scored predicted miRNA against *ESR 1 and ESR 2*.

### 2.5. Human Telomerase Reverse Transcriptase (hTERT)

Healthy cells are programmed for limited proliferative lifespan due to “telomere shortening”. During “telomere shortening” the telomere becomes shorter as the cell replicates and initiates cellular senescence signaling. A cell must deny the intracellular clock set by “telomere shortening” to acquire unlimited proliferation ability. Normally, the telomere length is maintained by the telomerase enzyme. Telomerase is composed of a catalytic subunit human telomerase reverse transcriptase (hTERT), an RNA subunit (hTR) human telomerase RNA (hTERC), and dyskerin [189,190]. hTERT constitutes the core functional unit of telomerase and contributes to the overall telomerase activity. The expression of hTERT is tightly regulated at the transcriptional level in normal somatic cells, whereas telomerase reactivation is a global molecular feature for nearly all (90%) human cancerous events [191]. Thus, overexpression of hTERT is a generic strategy a neoplastic cell develops to overcome restricted self-renewal and cellular senescence.

Though Hiyama et al., (1996) first reported telomerase activity in human breast tumors, Kirkpatrick et al., (2003) demonstrated a positive correlation between the *hTERT* mRNA expression and telomerase activity in human breast cancer [192,193]. The crucial cell signaling cascades dysregulated in cancer like c-MYC signaling, NF-κB signaling, TGF-β/Smad- signaling, Wnt/B-Catenin signaling converge to *hTERT* transcriptional upregulation [189,194]. hTERT reactivation in breast cancer is an early molecular event during neoplastic transformation that facilitates cell proliferation during tumorigenesis. Interestingly, hTERT reactivation enhances genomic instability [195,196]. One of the possible mechanisms behind telomerase-independent cancer cell proliferation in breast cancer from the ectopic hTERT expression was tRNA restoration [197]. While hTERT reactivation leads to an oncogenic effect, down-regulation of hTERT expression reverses the neoplastic phenotypes like unlimited proliferative lifespan and apoptosis evasion [198,199].

To date, a number of genetic and epigenetic mechanisms have been reported to upregulate hTERT during tumorigenesis. Both genetic and epigenetic regulations are observed within a ~260 base pair (bp) core promoter region extending −181 to +80 [200]. The 181-bp region just upstream to the transcription start site (TSS) is dense in transcription factor binding sites like SP1, AP2, c-Myc/Mad1, E2F1, HIF1, and ETS. There are binding sites for c-Myc/Mad1 and CTCF within the 80 bp segment downstream to the TSS. The genetic causes behind *hTERT* upregulation are promoter mutations, copy number variation and structural variants. About 1% of breast cancer cases have been detected with C228T and C250T mutations [201]. These *hTERT* promoter mutations introduce a new ETS binding motif where transcription factor GABP binds to lead to *hTERT* overexpression [202]. Approximately 25% of breast cancer patients show hTERT gain with increased *hTERT* mRNA expression [203]. Though reported in neuroblastoma, hTERT genomic rearrangements have not yet been reported in breast cancer.

**Figure 3:**
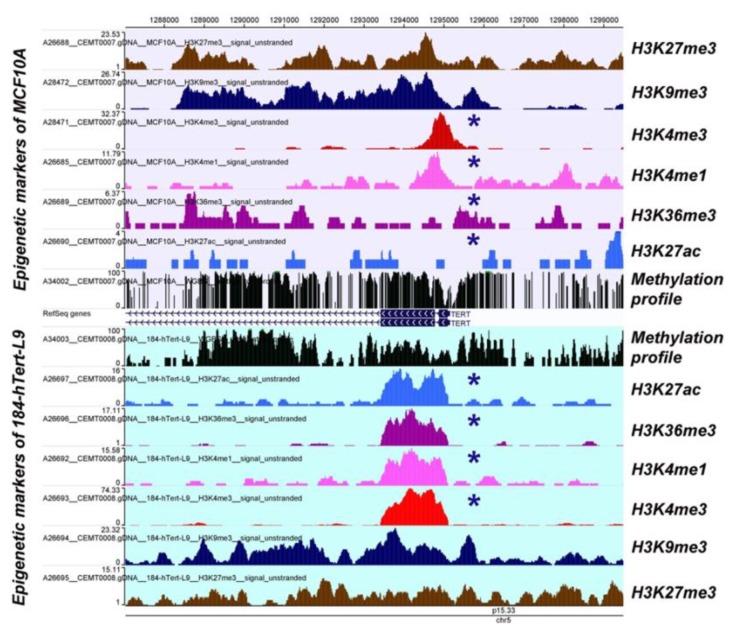
The histone modifications landscape of hTERT ectopic expression. Here, asterisk (*) denotes an apparently different modification mark. An overview of DNA methylation and histone modifications associated with *hTERT* ectopic expression is represented in two different hTERT-immortalized human mammary epithelial cell lines (MCF-10A cells and 184-hTERT-L9) [202]. Usually, the 184-hTERT-L9 cell line expresses hTERT more aggressively than the MCF10A cell line, whereas DNA methylation and histone modifications exhibited assorted patterns relatively exclusive to the cell type. Both of the cell lines show CpG methylation in the promoter and first exon. All the activating histone modification marks (H3K27ac, H3K36me3, H3K4me1, and H3K4me3) showed distinct patterns within the promoter region in terms of coverage and magnitude of deposition. Though the profile of repressive histone modification marks (H3K9me3 and H3K27me3) matched grossly, the magnitude of methyl-deposition was different for MCF10A cells and 184-hTERT-L9 cell line.

Covering about 70% of the breast cancer cases with *hTERT* reactivation, epigenetic regulations prevail over the genetic causes of *hTERT* re-expression. Ectopic *hTERT* expression may result from aberrant DNA methylation, histone modification or microRNA activity (Figure 3). Hypermethylation of the CpG sites within the *hTERT* promoter regulatory region paradoxically causes transcriptional activation. Mechanistically, hypomethylation enables transcription repressor CTCF binding and *hTERT* down-regulation. Bisulfite sequencing of a region from −650 to +150 suggested a consistent hypermethylation profile within the −650 to −400 region and a variable methylation pattern within −400 through +150 in multiple breast cancer cell lines [204]. Our lab showed a more comprehensive, quantitative comparison among *hTERT* promoter hypermethylation in breast cancer cell lines. We showed 25%, 82%, and 85% methylation of CpG sites (37) covering the *hTERT* regulatory region in MCF10A, MCF-7 and MDA-MB-231 cell lines, respectively [205]. Besides DNA methylation, *hTERT* expression is controlled by histone modification. The inhibition of the HDAC erasure of histone acetylation marks decreased *hTERT* expression in vascular smooth muscle cells [206]. Several empirical studies have reported not only deposition of activatory ac-H3, H3K9ac, and ac-H4 acetylation marks but also a decrease of repressive H3K9me3 and H3K27me3 marks on the *hTERT* promoter in breast, colon, and pancreatic cancer cell lines [64,207].

Another degree of *hTERT* reactivation is controlled by microRNAs. The microRNAs regulate *hTERT* expression by either directly targeting 3′-UTR/ORF of *hTERT* mRNA or indirectly interfering with other genes that control *hTERT* expression. A panel of miRNA like miRNA-1182, miRNA-532, miRNA-3064, miRNA-19b, miRNA-29a, and miRNA-661 inhibits tumorous cell growth and metastasis targeting *hTERT* [208,209,210,211,212]. Wu *et al.,* showed a direct effect of miRNA deregulation on *hTERT* expression in breast cancer cell lines MCF-7, T47D, BT-474, HCC1937, and MDA-MB-231. They reported miRNA-4458-mediated suppression of *hTERT* expression, whereas miRNA-4458 expression decreased in all studied breast cancer cell lines [80].

Besides direct control by microRNAs, hTERT expression could be controlled indirectly by microRNAs-mediated expressional regulation of other hTERT interacting genes. Feng et al., (2017) showed that miRNA-138 mimic attenuates hTERT and K17 expressions simultaneously in HaCaT cells. They suggested that miRNA-138 lowers the expression of K17 protein which in turn reduces hTERT expression [213]. MiRNA-4458 down-regulates *CPSF4* expression targeting 3′-UTR that results in the reduction of hTERT expression [80]. However, some indirectly acting miRNAs control the cellular hTERT level positively. For example, miRNA-21 reduces PTEN level in order to induce the expression of hTERT through PTEN/ERK1/2 signaling pathway [214].

miRNAs may exploit diverse strategies to control hTERT expression. Conventional positive and negative miRNAs regulators of hTERT exert their effect through molecular interaction. Beyond typical regulatory paradigms, hTERT expression has been reported to be controlled by cis-acting elements/genomic elements. MiRNA-615-3p down-regulates *hTERT* expression interfering with the long-range interaction between *hTERT* promoter and its distal enhancer. Indeed, the expression of the *HOXC5* gene is supplemented by the expression of miRNA-615 as the *miRNA-615-3p* gene locates within intron 1 of the *HOXC5* gene. HoxC5, in turn, recruits Pbx4 to form the HoxC5:Pbx4 complex that binds to HDAC1 and/or HDAC3 in order to repress *hTERT* expression [215].

**Figure 4:**
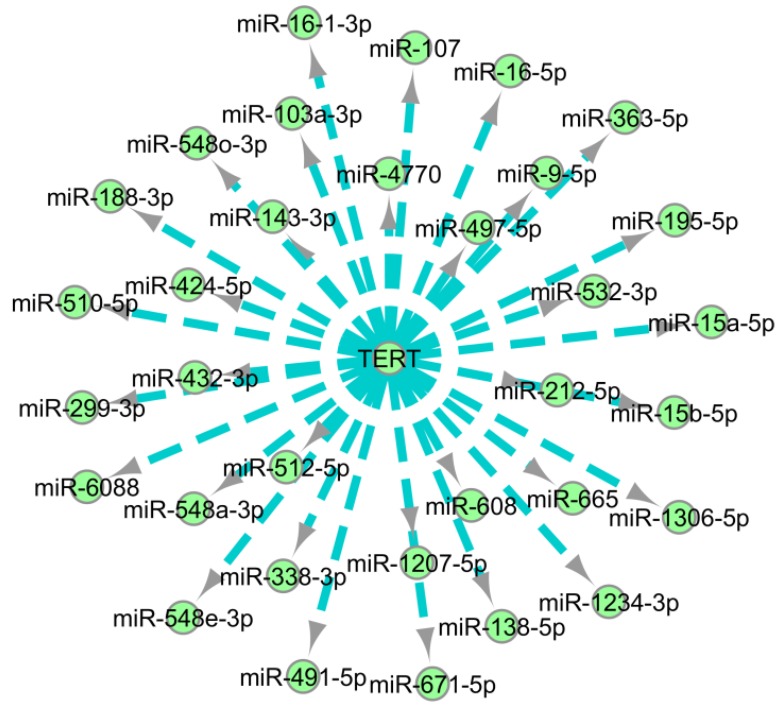
Interaction network of predicted miRNAs against TERT. The network was constructed using Cytoscape taking predicted miRNAs with a high score from mirDIP [187,216]. During network building, predicted miRNAs were targeted to interact with TERT. The length between the TERT and a subject miRNA indicates an integrated prediction score.

Similar to estrogen receptors, the list of hTERT targeting miRNAs is growing. As hTERT is a potential therapeutic target for cancer drug discovery, miRNAs that influence the molecular and physiological role of hTERT are crucially important. Besides empirical studies, computational tools could provide useful information about the miRNAs candidates against hTERT. A set of predicted miRNAs against TERT has been depicted in Figure 4. One of the major concerns about hTERT miRNA prediction is the target overlapping with other genes, while the accuracy of miRNA prediction is largely experimental.

## 3. Epigenetic Version of Breast Cancer Hallmarks: An Avenue to Reversibility

Similar to other cancers, breast cancer oncogenesis is a multistep process directed to acquire cancer hallmarks: Constant proliferative signal, insensitivity to growth-suppressing signal, apoptosis evasion, unlimited replicative lifespan, sustained angiogenesis and invasion and metastasis ability [217]. Early non-lethal genetic mutations during neoplastic transformation alter genomic homeostasis to cause tumorigenic mutations that propel a normal cell to proliferate spontaneously and escape programmed cell death/apoptosis. Additional mutations establish oncogenic homeostasis that pushes the neoplastically transforming cell through a clonal diversification route [218]. Sequentially, the neoplastic cell invades the surrounding tissue and induces sustained angiogenesis as a non-metastatic tumor. The non-metastatic tumor usually metastasizes to a secondary site after the loss of adhesiveness, immune evasion, and diversification. Therefore, breast cancer is largely a genetic disorder according to the concept of the “cancer hallmarks” [219].

**Figure 5:**
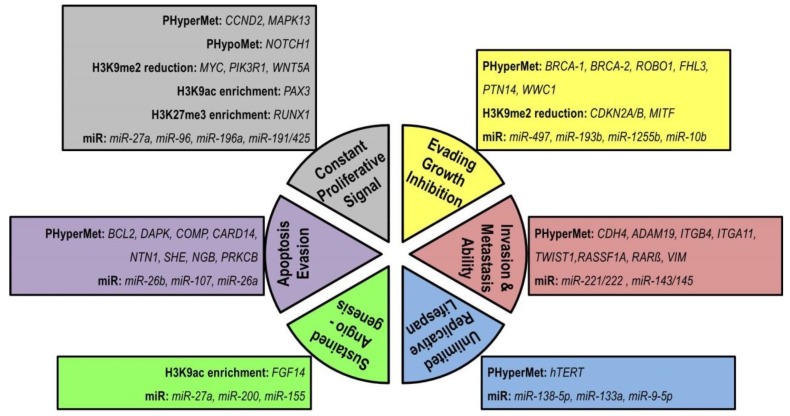
The epigenetic changes associated with breast cancer hallmarks. Here, PHyperMet means promoter hypermethylation, PHypoMet means promoter hypomethylation, miR means microRNA. Every single cancer hallmark could be controlled by an epigenetic regulation alone or in coordination with genetic regulation. Besides the epigenetic alteration of oncogene and tumor suppressor gene regulation [25,30,52,53], microRNAs have been attributed to different oncogenic hallmarks [13,61] of breast cancer.

Though genomic instability constitutes the foundation of the breast cancer hallmarks, loss of epigenetic homeostasis can sustain cancer hallmarks (Figure 5). Epigenetic dysregulation of a significant number of oncogenes and tumor suppressor genes have been reported over the last few decades [220]. Indeed, epigenetic alterations appear to be more extensive compared to genetic alterations [221].

Each of the hallmarks of breast cancer could be achieved by a coordinated alteration of genetic and epigenetic regulation [222]. If not the initial steps, the later stages of breast cancer are crucially governed by epigenetic regulation [223]. The reversibility of epigenetic regulation challenges the efficiency of the epigenetic mechanism but gradual epigenetic evolution over the long period of tumor latency allows the acquisition of the traits required for the cancer hallmarks [224]. Considering the therapeutic goal of breast cancer, the reversibility of the epigenetic event associated with breast cancer opens new avenues for breast cancer therapeutic intervention through the reversal of breast cancer hallmarks.

## 4. Epigenetics Strategies of Breast Cancer Reversal

By this point, we have seen an account of genetic and epigenetic factors involved with breast cancer. Mutation, copy number variation, or genetic rearrangement lead to breast tumorigenesis, while epigenetic processes include DNA methylation, histone modification, and microRNAs. As aforementioned, genetic alterations are mostly unidirectional through epigenetic changes are usually reversible and plastic [220]. Therefore, targeting epigenetic processes to alter their role in tumorigenesis and/or malignancy could be a promising therapeutic strategy to treat or prevent breast cancer.

### 4.1. Epigenetic Drugs

Epigenetic drugs interfere with the “epigenetic machineries” to counteract the tumorigenic outcome of the concerned epigenetic process. Epigenetic drugs target either the reader or the eraser of the epigenetic marks like DNA methylation, histone acetylation, histone methylation, etc. Additionally, the microRNAs are targeted directly through nucleic acid-based drugs [225]. The DNMT inhibitors are a class of epigenetic drugs that block DNMTs (DNMT1, DNMT3A, and DNMT3B) activity and subsequent DNA methylation. A number of epigenetic drugs inhibit HATs and HDACs to regulate histone acetylation. The HDMs and HMTs are two common classes of enzymes involved with histone methylation and these are frequently targeted by epigenetic drugs. In the case of miRNAs, tumor suppressor miRNAs that are down-regulated during tumorigenesis can be supplemented with miRNA mimics through miRNA replacement therapy. On the other hand, anti-miRNA therapy blocks or traps the oncogenic miRNAs either delivering miRNA antagonist complementary to the target miRNA or decoying the target miRNA with sponge RNAs that harbor complementary binding sites for the target miRNA [226].

**Figure 6:**
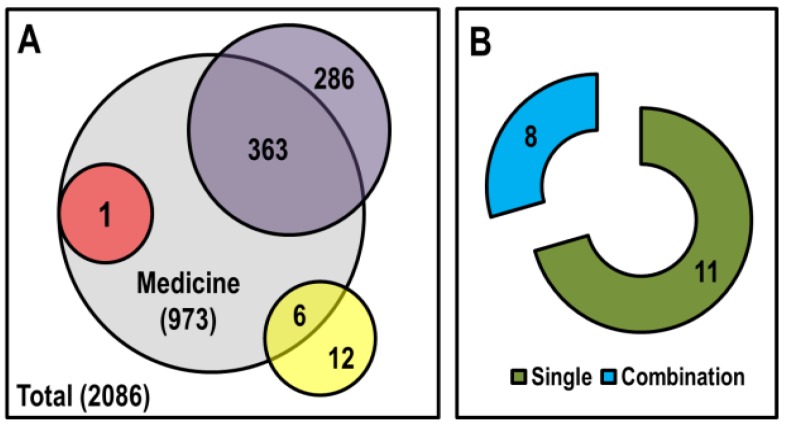
Comparison of clinical trials completed for chemotherapeutic and epigenetic drugs. (**A**) A summary of the accomplished clinical trials on breast cancer from 2001. Here, the ash circle represents medicines that reached phase II clinical trials, the purple circle represents chemotherapeutic drugs, the pink circle represents DNMT inhibitors, the yellow circle represents HDAC inhibitors and the white rectangle represents the total number of clinical trials on breast cancer from 2001. (**B**) The status of the epigenetic drugs that have undergone complete clinical trials for breast cancer therapy from 2001. Here, the green crescent represents combination cases when the epigenetic drug was applied with other chemotherapeutic drugs/targeted therapy and the blue crescent represents epigenetic drugs applied alone. The data were collected from ClinicalTrials.gov [226].

In contrast to the conventional chemotherapeutic drugs, epigenetic drugs against breast cancer are limited (Figure 6 and Table 4). Prospects of epigenetic drugs for breast cancer rely on several features. Firstly, 8 out of 10 women develop breast cancer without any previous family history. This sporadic nature of breast cancer implies acquired genetic alterations toward tumorigenesis as well as more epigenetic interplay toward acquired phenotypes. Secondly, epigenetic regulations either regulate or facilitate the tumorigenesis-malignancy-metastasis axis of cancer signaling. Epigenetic processes have been reported to control a panel of genes involved in tumorigenesis, migration, invasion, and metastasis of breast cancer. Thirdly, reversibility is a feature common to epigenetic regulation and cancerous cellular processes. For example, substantial evidence suggests that epithelial-to-mesenchymal transformation is likely regulated solely through a revocable epigenetic switch [227]. Though it is uncertain how much research with epigenetic drugs against breast cancer will expand in the near future, epigenetic drugs must have at least a facilitating role toward breast cancer regression/preventive therapy.

### 4.2. Epigenetic Diet

The epigenetic mechanisms of a cell are resilient to environmental stimuli leading to a different epigenome orientation from the same genomic background [231]. Diet is one of the key epigenetic stimuli that instigate epigenetic reprogramming [232]. Phytochemicals found in dietary fruits and vegetables interact with epigenetic machineries to alter the ongoing epigenome status. Not all diets have the same influence on the epigenome makeup of the cells. We coined the term “epigenetic diet” in 2011 to recognize diets with prominent effects on epigenetic processes [233]. Over the last two decades, the epigenome reshaping capability of epigenetic diets has drawn attention as an alternative, preventive, and safe tool for dietary management of a number of diseases.

Dietary bioactive components are promiscuous in their target selection. All the major enzyme classes within the DNA methylation-histone modification loop are targeted by the epigenetic diets [234] and the exact mechanisms the dietary components apply to regulate gene expression are mostly well-defined. Many of these mechanisms were derived from studies on the anticancer effect of dietary bioactives [235]. With a few exceptions, epigenetic diets affect transcriptional regulation of target genes inhibiting DNMTs, HMTs, HDACs, and chromatin remodeling complexes. Interestingly, epigenetic diets exhibit a bivalent role toward miRNAs expression depending on the nature of the target miRNAs [235,236].

**Figure 7:**
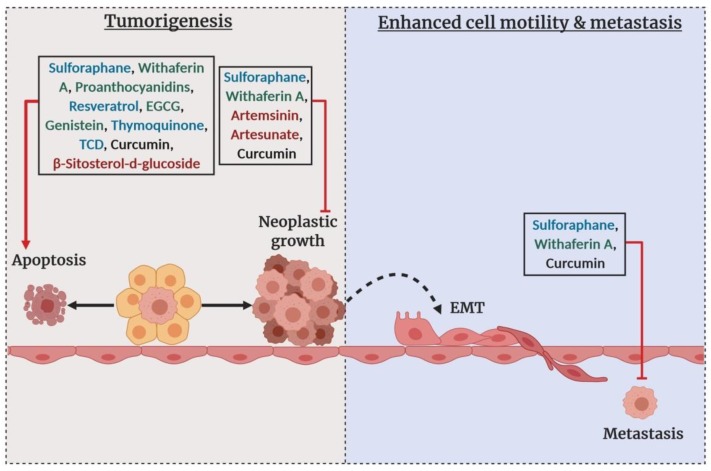
The effect of epigenetic phytochemicals on breast cancer. Here, blue color phytochemicals indicate primarily HDAC inhibitors, green color phytochemicals indicate DNMT inhibitors, red color phytochemicals indicate miRNA profile modulators and black color phytochemicals indicate global epigenetics modulators. During tumorigenesis, cells undergo neoplastic transformation to favor uncontrolled neoplastic growth avoiding cell cycle regulation and apoptosis. As the cells proceed to malignancy, the neoplastic cells become motile through epithelial–mesenchymal transition [EMT], invade the surrounding tissue and metastasize to the distal anatomic location. The epigenetic phytochemicals interfere with tumorigenesis to either enhance apoptosis or prevent neoplastic growth. A number of epigenetic phytochemicals also halt the metastasis.

The epigenetic reprogramming brought by the dietary phytochemicals modulates the fate of a cancerous cell. A number of preclinical in vitro and in vivo studies highlighted the effect of epigenetics diets on different molecular features of breast cancer (Figure 7). The epigenetic diets shape the epigenome interacting directly or indirectly with DNA methylation, histone modifications, and miRNAs expression profiles. The epigenetic modulatory phytochemicals often alter DNA methylation and histone modification marks to change the expression of a set of genes from different cellular pathways like cell cycle, apoptosis, tumor suppression, DNA repair, cell adhesion, chromatin remodeling, etc. Phytochemicals such as withaferin A, sulforaphane (SFN) and curcumin have global effects through the tumorigenesis-metastasis pathways [237,238,239,240,241]. A number of other phytochemicals including thymoquinone, 3β,7β,25-trihydroxycucurbita-5,23(*E*)-dien-19-al (TCD), β-Sitosterol-d-glucoside, artemisinin, artesunate, proanthocyanidins, and resveratrol are reported newly as epigenetic modulators of breast cancer [242,243,244,245,246]. Interestingly, the epigenetic role of a particular phytochemical is sometimes multifaceted. For example, Lewinska et al., (2017) reported that sulforaphane, a well-known HDAC inhibitor, has an inhibitory effect on DNMTs. The same group, additionally, reported the change of at least 36 different microRNAs expression profiles including miRNA-23b, miRNA-92b, miRNA-381, and miRNA-382 as an outcome of SFN treatment of breast cancer cells (MCF-7, MDA-MB-231, and SK-BR-3) [247].

Beyond the arena of basic research, epigenetic diets also show promise as medicinal and chemopreventive tools at the interventional level. The green tea extract component DNMT inhibitor EGCG and Polyphenon E were used in two different phase 2 clinical trials (NCT00917735 and NCT00676793) to investigate the effect of green tea extract on breast cancer progression. In another randomized phase I trial (NCT00516243), green tea catechin extract was administered to women with hormone receptor-negative stage I-III breast cancer. A phase 2 clinical trial (NCT00843167) of broccoli sprout extract, a cruciferous vegetable rich in HDAC inhibitor sulforaphane (SFN), has been completed recently for treating ductal carcinoma in situ and/or atypical ductal hyperplasia [236]. At least three different clinical trials with DNMT inhibitors genistein (NCT00244933, NCT00290758, NCT00099008) and HDAC inhibitor curcumin (NCT01042938, NCT01740323, NCT02556632) have been completed on breast cancer patients. Besides these studies, a number of clinical trials with epigenetic diets are either ongoing or beginning [226]. Most interestingly, some of these epigenetic bioactives are acquiring focus through pharmaceutical developments that provide new hope with respect to epigenetic diets. A very recent example is alpha-cyclodextrin encapsulated sulforaphane (SFX-01) which is now under interventional study (NCT02970682) against metastatic breast cancer [248].

### 4.3. Epigenome Editing Tools

From the biochemical point of view, epigenetic regulation primarily involves chemical tagging of the DNA and associated histone proteins through DNA methylation, histone acetylation, and histone methylation. The reversibility of these chemical modifications enables the editing feasible using “designed modular tools”. The basic structure of editing tools includes a programmable DNA-binding domain and an epigenetic effector domain of interest. Three different programmable DNA-recognition domains have been reported widely in epigenome editing, including the zinc finger (ZF), transcription activator-like effector (TALE), and nuclease-deficient Cas9 (dCas9) [249]. The epigenetic effectors are rather diverse ranging from the reader and erasure of DNA and histone modification marks as well as artificial transcription factors [250].

Each ZF domain consists of ~30 amino acids and recognizes a particular set of three to four base pair sequences in the major groove of DNA. Synthetically, ZF proteins (ZFPs) are crafted to contain four to six zinc finger domains arranged in tandem to specifically target a stretch of 18 base pairs [250,251]. The DNA-binding domains of the transcription activator-like effectors (TALEs) are an array of ~34 amino acid repeat domains each of which recognizes a single base pair by using two hypervariable residues at positions 12 and 13 [250,251]. In the case of dCas9, a point mutation in endonuclease domains creates a catalytically dead version of RNA-guided DNA endonucleases Cas9 that binds its guide RNA and can recognize a DNA strand complementary to the guide RNA sequence [252]. Thus, ZFN or TALEN involve protein-based DNA targeting, whereas CRISPR-dCas9 involves small RNA-mediated sequence targeting [253]. As a result, targeting a desired sequence is more comprehensive and laborious with ZFN or TALEN techniques, while Cas9 targeting requires only a suitable guide RNA [254].

Epigenetic editing of breast cancer is relatively new compared to the other cancer types. Epigenetic editing of breast cancer began with classical epigenome editing tools ZFPs and TALEs. One of the early attempts was made by Rivenbark et al., in 2012. They down-regulated Maspin and SOX2 expression in SUM159 and MCF-7 breast cancer cell lines using a synthetic epigenetic construct of six ZF domain (6ZF) array linked to the DNMT3a catalytic domain (6ZF-DNMT3a). The downregulation of tumor suppressor Maspin promoted anchorage-independent growth of breast cancer, whereas suppression of SOX2 oncogenes reduced breast cancer cell proliferation [255]. Targeting the *SOX2* promoter with 6ZF- KRAB (Krüppel-associated box domain) repressed SOX2 expression and reduced cell proliferation and colony formation of MDA-MB-435s and MCF-7 cells [256]. In another study, the *Her2/neu* promoter was targeted with ZF-G9a-induced H3K9me2 and suppression of Her2/neu expression concomitant with cell growth inhibition [257]. Transcription activator VP64 bearing TALE construct (TALE-99 VP64) upregulated the MASPIN expression in MCF-7 cells [258].

The simplicity, specificity, and flexibility of clustered regularly interspaced short palindromic repeats (CRISPR)-dCas9 circuit has made the CRISPR-dCas9-based epigenome editing an alternative choice for epigenome editing [259]. The CRISPR-dCas9-based epigenome editing tool is a promising tool for both pathogenesis and therapeutic research. Saunderson et al., targeted *CDKN2A*, *RASSF1*, *HIC1,* and *PTEN* genes using a dCas9-DNMT3A construct in normal primary myoepithelial cells. The ultimate result was promoter hypermethylation and consequential gene silencing and suppression of cell senescence [259]. In another study, targeting Par-4 with dCas9-p300 increased H3K9 and H3K27 acetylation at the *Par-4* promoter with consequential reexpression of Par-4 protein both at the transcriptional and translational level in the recurrent breast cancer. This reactivation of the Par-4 was associated with resensitized recurrent tumor cells to a microtubule-targeting chemotherapeutic drug [260]. Besides the epigenetic effector, CRISPR-dCas9 base target recognition could be optimized to modulate the epigenetic regulation in breast cancer. For example, Moses et al., exploited the dCas9-VPR construct to target the tumor suppressor *PTEN* promoter in SUM159 TNBC cell lines, which led to the PTEN expressional upregulation. The PTEN activation, in turn, inhibits the oncogenic PI3K/AKT/mTOR-signaling axis and MAPK oncogenic signaling pathways [261].

### 4.4. miRNA-Based Therapy

Dysregulation of miRNA expression is a molecular signature generic to breast cancer. MiRNAs have both oncogenic and tumor suppressor roles during breast tumorigenesis [262]. Like other oncogenes or tumor suppressor genes, miRNAs are considered as a potential breast cancer therapeutic target. MiRNA-based treatments rely either on inhibition of oncogenic miRNAs or restoration of tumor suppressor miRNAs [263]. The miRNA therapies are largely nucleic acid-based in nature. Practically, chemically modified, synthetic, stable and functional nucleic acids are used to supplement or reduce the miRNA of interest during miRNA-based therapy [224].

#### 4.4.1. MiRNA Mimic

MiRNA mimics are synthetic oligonucleotides analogous to miRNA. From a therapeutic standpoint, tumor-suppressive miRNAs are usually targeted for miRNA mimics delivery. These miRNA mimics may be modified chemically for better cellular half-life and improved delivery and can restore the tumor-suppressive miRNAs that are downregulated in breast cancer cells [263]. Similar to miRNAs, miRNA mimics bind to and destabilize the mRNA expression of the oncogenes/oncogenic signaling pathway. To date, a number of investigational miRNA mimics have been reported against breast cancer and are summarized in Table 5. Functionally, miRNA mimics interfere with different breast cancer hallmarks like cell proliferation, migration, invasion, apoptosis, etc.

#### 4.4.2. MiRNA Antagonists (antagomiRs)

AntagomiRs are synthetic anti-sense oligonucleotides that antagonize a target miRNA of oncogenic nature. AntagomiRs binds to a target oncogenic miRNA through base complimentary and inhibits the functionality of target miRNA disrupting miRNA-mRNA interaction [224]. Usually, antagomiRs are similar to the target miRNA in length. The ultimate result is a functional reduction of oncogenic miRNA within the cell. However, the use of antagomiRs to combat breast cancer is in its nascent investigational stages.

In terms of affecting cancer hallmarks, antagomiRs play a diverse role. Studies indicate that antagomiRs could be exploited against breast cancer with very specific purposes because antagomiRs may exhibit cancer hallmark-specific functionality. For example, anti-miR-135a was effective against metastasis, whereas anti-miRNA-492 inhibited growth [268,269]. However, other reports suggest a more global role of antagomiRs such as anti-miRNA-203 and anti-miRNA-937. Anti-miRNA-203 resisted tumor growth and metastasis, while anti-miRNA-937 prevented proliferation and induces apoptosis of breast cancer [270,271].

##### Anti-miRNA-135a

MiRNA-135a is an oncogenic miRNA that selectively facilitates breast cancer cell migration and invasion. MiRNA-135a is highly expressed in metastatic breast tumors. At the molecular level, miRNA-135a targets metastasis suppressor HOXA10 and enhances cancerous metastasis. In a study, Chen et al., (2012) suppressed cell migration and metastatic potential of the highly invasive BT549 cell line by transfecting the cell line with anti-miRNA-135a [268].

##### Anti-miRNA-492

MiRNA expression profiling suggests that miRNA-492 is overexpressed in breast cancer tissues and several breast cancer cell lines including BT549, MCF-7, Bcap37, SKBR3, ZR75-30, T47D, MDA-MB231, MDA-MB453, and MDAMB435. The sex-determining region Y-box 7 (SOX7) is a direct target of miRNA-492. Down-regulation of *SOX7* by miRNA-492 subsequently enhances G1-S transition through upregulating *cyclin D1*, *c-Myc* expression and Rb phosphorylation. Thus, supplementing miRNA-492 with miRNA-492 mimics stimulates greater proliferation and anchorage-independent growth of breast cancer cells. On the contrary, treating breast cancer cells with anti-miRNA-492 results in cell proliferation arrest [269].

##### Anti-miRNA-203

The cancer prompting role of miRNA-203 is exerted through fibroblast growth factor 2 (*FGF2*), a direct target of miRNA-203. Under normal expressional level, FGF2 inhibits the TGF-β pathway that promotes tumor cell growth and migration. The study of He et al., (2016) demonstrated a marked overexpression of miRNA-203 in clinical breast cancer specimens compared with normal breast tissues and in the MCF-7 cell line. Knocking down miRNA-203 with anti-miRNA-203 counteracts MCF-7 cell proliferation and migration. Thus, anti-miRNA-203 rescues the miRNA-203-mediated activation of the TGF-β pathway along with proliferation and migration of breast cancer cells [270].

##### Anti-miRNA-937

MiRNA-937 is a highly expressed miRNA in the MCF-7 breast cancer cell line. Clinically, miRNA-937 expression is inversely correlated with the survival rate of breast cancer patients. While miRNA-937 mimics induce proliferation, anti-miRNA-937 promotes apoptotic cell death and G1/S phase-arrest of MCF-7 cells. In the MCF-7 cell line, miRNA-937 targets APAF1 at the 3′UTR to destabilize the *APAF1* expression. As APAF1 overexpression stimulates apoptosis and cell proliferation arrest, anti-miRNA-937 supports inhibition of breast cancer growth knocking down the miRNA-937 [271].

#### 4.4.3. MiRNA Sponges

MiRNA sponges are synthetic nucleotides that decoy oncogenic miRNA to reduce the functional abundance of miRNA within the cell. MiRNA sponges compete with mRNA for binding to the target miRNA [61]. Once trapped by miRNA sponges, target miRNAs forgo interaction with target mRNAs. MiRNA sponges are functionally similar to antagomiRs except for the difference in the size. Usually, miRNA sponges harbor multiple sites in tandem for target miRNA [263].

##### miRNA-933 Sponge

Breast cancer tissue, as well as breast cancer cell lines such as MCF-7, MDA-MB-231, MDA-MB-435, T47D, and SKBR-3, showed a significant down-regulation of TFAP2A-AS1 expression. The lncRNA TFAP2A-AS1 is an endogamous sponge against miRNA-933. TFAP2A-AS1 overexpressing cell lines showed enhanced cell cycle arrest and apoptosis. MCF-7 and MDA-MB-231 cells transfected with TFAP2A-AS1 undergo significant down-regulation of CDK6, cyclin D1, and cyclin E1 that lead to cell cycle arrest. Conversely, SMAD2 expression is induced by TFAP2A-AS1 as *SMAD2* is directly targeted by miRNA-933. SMAD2 is a component of the TGF-β/SMAD signaling pathway involved in apoptosis and cell cycle control [272].

##### miRNA-23a Sponge

LncRNA GAS5 is a long noncoding RNA that functions as a miRNA-23a sponge. Expressional analysis of primary breast cancer tissue and breast cancer cell lines (i.e., MDA-MB-23l, MDAMB-453, BT549, SK-BR-3, and MCF-7) demonstrated down-regulation of *GAS5* expression. Ectopic expression of GAS5 in MDA-MB-23l promoted autophagy. At the molecular level, GAS5 expression releases miRNA-23a-mediated attenuation of autophagy-related proteins 3 (ATG3) expression. Thus, LncRNA GAS5 functions as a tumor-suppressive sponge through the GAS5-miRNA-23a-ATG3 axis to control the autophagy pathway [273].

##### miRNA-10b Sponge

MDA-MB-231 is a breast cancer cell line with higher metastatic potential that shows higher expression of miRNA-10b which has been speculated to be linked with breast cancer metastasis. When MDA-MB-231 cells are transfected with a sponge plasmid against miRNA-10b, transfected MDA-MB-231 cell line showed a reduction in migration and invasion capability besides reduced cellular growth. MiRNA-10b knockdown with miRNA-10b sponge upregulates *HOXD-10* that, in turn, inhibits breast cancer metastasis [274].

##### miRNA-203a-3p Sponge

MiRNA-203a-3p upregulation is associated with enhanced colony formation, migration and poor breast cancer prognosis. MiRNA-203a-3p targets *SOCS3* which inhibits breast cancer cell proliferation. Circular RNA circTADA2A-E6 acts as miRNA-203a-3p sponge. CircTADA2A-E6 restores the cellular level of SOCS3 to the normal that is frequently downregulated in breast cancer. Thus, CircTADA2A-E6 mediates miRNA-203a-3p/SOCS3-assisted inhibition of proliferation, migration, and invasion of breast cancer [275].

##### miRNA-21 Sponge

MCF-7 is characterized by higher expression of miRNA-21. Transfecting MCF-7 cell line with miRNA-21 sponge plasmid significantly decreases the miRNA-21 expression. This knockdown leads to decreased cell viability from G1 phase arrest as well as caspase-3 apoptotic pathway activation. Additionally, miRNA-21 sponge increased the responsiveness of MCF-7 cells to anti-tumor drugs such as doxorubicin and cisplatin [276].

## 5. Conclusions

The epigenetic basis of breast cancer has come into light through many investigations over the last two decades. Soon after this identification, epigenetic processes such as DNA methylation, histone modification, and miRNA regulations became targets for altering the course of breast cancer with positive and promising outcomes. Still, a more comprehensive base of research is needed for targeting epigenetic regulation as a feasible and effective treatment option against breast cancer. Though we have a gross overview of the breast cancer-specific epigenomic landscape, more studies should focus on the epigenome status to deduce a high-resolution, comprehensive epigenomic map incorporating miRNA regulations besides DNA and histone modifications. We need to characterize the epigenomic behavior of potential genes of interest and associated epistatic plasticity. To be considered as drugable, epigenetic targets should be studied more in terms of pharmacodynamics and efficacy.

Epigenetic instability plays a crucial role in breast cancer initiation and progression. Being extensive and reversible, the epigenetic homeostasis associated with breast cancer demands more intense attention in future investigations. Several attempts to reverse the breast cancer hallmarks by targeting the epigenetic machinery have proven successful and encouraging. Epigenetic drugs, diets and, synthetic tools are, in this regard, a flourishing scope for future research directions with breast cancer.

With convincing promise toward breast cancer therapy, epigenetic editing tools demand more basic investigations for demonstrating effectiveness and efficacy. Editing circuits such as ZFPs, TALEs, and (CRISPR)-dCas9 should be proven safe to be used under pre-clinical and clinical setup. Though the miRNA candidates for therapeutics development are growing rapidly, little is known about all possible biological interactions of a candidate miRNA. The biofunctionality of miRNAs should be more detailed as several existing data allude noncanonical mechanisms of miRNA action. Additionally, we need to address a number of challenges associated with miRNA-based therapeutics development, including target overlapping, selective drug delivery, and biological stability.

## Figures and Tables

**Table 1:** The miRNAs involved with breast cancer.

MicroRNA Name	Target Gene/Signaling Pathway	Effect on Breast Cancer Process							References
		Proliferation and/or Growth	Apoptosis	Migration	Invasion	EMT	Angiogenesis	Metastasis	
miR-3614	*TRIM25*	*↓							[72]
miR-454-3p	Wnt/β-catenin signaling							↑	[73]
miR-216a	Wnt/β-catenin signaling	↓		↓					[74]
miR-4458	*SOCS1*	↓	↑						[75]
miR-140-3p	*TRIM28*	↓		↓					[76]
miR-483	*SOX3*	↓							[77]
miR-214	*α1-AT*; PI3K/Akt/mTOR signaling			↑	↑				[78]
miR-216a-5p	*PAK2*	↓						↓	[79]
miR-4458	*CPSF4*	↓			↓				[80]
miR-653	ZEB2 signaling	↓	↑						[81]
miR-190	AKT-ERK signaling						↓	↓	[82]
miR-370	WNK2	↑							[83]
miR-193a-3p	GRB7	↓		↓	↓				[84]
miR-591	*TCF4*; Hippo-YAP/TAZ signaling	↑			↑				[85]
miR-153	*RUNX2*	↓		↓	↓	↓			[86]
miR-4513	*TRIM3*	↑		↑	↑				[87]
miR-216a	*PKCα*		↑						[88]
miR-374c-5p	*DEPDC1*	↓	↑	↓		↓			[89]
miR-890	*CD147*	↓	↑	↓					[90]
miR-142-3p	*ESR1*	↓	↑						[91]
miR-449b-5p	Wnt/β-catenin signaling	↓			↓				[92]
miR-135	Wnt/β-catenin signaling	↓		↓	↓	↓			[93]
miR-1287-5p	*PIK3CB*	↓							[94]
miR-30a	*Snail*	↓			↓				[95]
miR-135b	*APC*	↑						↑	[96]
miR-124	ZEB2 signaling				↓			↓	[97]
miR-4282	*Myc*	↓			↓			↓	[98]
miR-3196	ERBB3		↑						[99]
miR-1179	Notch signaling							↓	[100]
miR-590-3P	sirtuin-1		↑						[101]
miR-645	uPA				↓				[102]
miR-99a-5p	CDC25A	↓	↑		↓				[103]
miR-196b-5p	COL1A1	↑						↑	[104]
miR-421	PDCD4	↓	↑	↓	↓				[105]
miR-508-3p	ZEB1				↓	↓			[106]
miR-3178	Notch1	↓						↓	[107]
miR-340-5p	LGR5; Wnt/β-catenin signaling	↓							[108]
miR-511	SOX9; PI3K/Akt pathway	↓	↑					↓	[109]
miR-424	CDK1	↓							[110]
miR-301b	CYLD	↑	↑						[111]
miR-194-5p	Wnt/β-catenin signaling	↓		↓	↓				[112]
miR-199b-5p	DDR1	↓		↓	↓				[113]
miR-1247-5p	DVL1/Wnt/β-catenin signaling	↓							[114]
miR-628	SOS1			↓	↓				[115]
miR-590-5p	Wnt-β-catenin signaling			↓	↓	↓			[116]
miR-483-3p	cyclin E1	↓							[117]
miR-125b-5p	KIAA1522	↓		↓	↓				[118]
miR-1254	RASSF9	↑	↓						[119]
miR-590-3p	ATF3	↓	↑						[120]
miR-125a-5p	BAP1	↓	↑						[121]
miR-1284	ZIC2	↓			↓				[122]
miR-92b	EZH2	↓			↓				[123]
miR-140-5p	Wnt1	↓							[124]
miR-185-5p	RAGE					↓			[125]
miR-320a	IGF-1R	↓			↓				[126]
miR-498	PTEN	↑		↑					[127]
miR-202	KRAS	↓		↓	↓				[128]
miR-1301-3p	ICT1	↓							[129]
miR-129-5p	CBX4	↓							[130]
miR-361-5p	RQCD1; EGFR/PI3K/Akt pathway			↓	↓				[131]
miR-433	Rap1a; MAPK signaling	↓							[132]
miR-20a-5p	RUNX3	↑							[133]
miR-1271	SPIN1	↓							[134]
miR-130a-3p	RAB5B			↓	↓				[135]
miR-384	ACVR1	↓		↓					[136]
miR-30a	ROR1					↓		↓	[137]
miR-449a	PLAGL2			↓	↓				[138]
miR-328-5p	RAGE	↓							[139]
miR-708-3p	ZEB1, CDH2 and vimentin							↓	[140]
miR-144	CEP55	↓		↓	↓				[141]
miR-433	AKT3	↓							[142]
miR-1204	VDR	↓				↑		↑	[143]
miR-424-5p	DCLK1	↓		↓	↓				[144]
miR-194	Fbxw-7	↑							[145]
miR-577	Rab25					↓		↓	[146]
miR-190	SMAD2							↓	[147]
miR-664	IRS1	↓			↓				[148]
miR-320	AQP1	↓		↓	↓				[149]
miR-519d	MMP3	↓		↓	↓				[150]
miR-8084	ING2	↑	↓			↑			[151]
miR-372	LATS2	↑							[152]
miR-19b-1	VEGF	↓					↓		[153]
miR-124-3p	PDCD6							↓	[154]
miR-130a	FOSL1			↓	↓				[155]
miR-770	STMN1			↓	↓				[156]
miR-25-3p	BTG2	↑							[157]
miR-3188	TUSC5; p38-MAPK signaling	↑	↓	↑					[158]

*↑ indicates promotion/induction, ↓ indicates reduction/inhibition, ‘miR’ means microRNA.

**Table 2:** The miRNA expression profile in different breast tumor subtypes.

Breast Tumor Subtypes	MiRNA Expression Profile	
	Upregulated miRNAs	Downregulated miRNAs
Luminal A	miR-126, miR-136, miR-100, miR-99a, miR-145, miR-10a, miR-199a, miR-199b, miR-130a, miR-30a-3p, miR-30a-5p, miR-224, miR-214, let-7a*, let-7b*, *let7c*, *let-7f*, miR-342*	miR-150, miR-142-3p, miR-142-5p, miR-106a, miR-106b, miR-18a, miR-93, miR-25, miR-187, miR-135b
Luminal B	miR-106b, miR-93, miR-25, miR-10a, miR-30a-3p, miR-30a-5p, miR-224, *let-7f*	miR-150*, miR-142-3p, miR-142-5p*, miR-148a, miR-18a, miR-155*, miR-187, miR-135b, miR-126, miR-136, miR-100, miR-99a, miR-145, miR-130a
Basal-like	miR-150, miR-142-3p, miR-142-5p, miR-148a, miR-106a*, miR-106b, miR-18a*, miR-93, miR-155*, miR-25, miR-187, miR-135b*	miR-126, miR-136, miR-100, miR-99a, miR-145, miR-10a, miR-199a, miR-199b, miR-130a, miR-30a-3p, miR-30a-5p, miR-224, miR-214, *let-7a*, *let-7b*, *let7c*, *let-7f*, miR-342
HER2^+^	miR-150, miR-142-3p, miR-142-5p, miR-148a, miR-106b, miR-25, miR-187*	miR-106a, miR-18a, miR-93, miR-155, miR-135b, miR-126, miR-136, miR-100, miR-99a, miR-145, miR-10a, miR-199b, miR-130a*, miR-30a-3p*, miR-30a-5p*, miR-224*, *let-7a*, *let-7b*, *let7c*, *let-7f*, miR-342
Normal-like	miR-135b, miR-126*, miR-136, miR-100, miR-99a, miR-145, miR-10a, miR-199a, miR-199b, miR-130a*, miR-30a-3p, miR-214, *let7c*	miR-142-3p, miR-148a, miR-106a, miR-106b*, miR-93*, miR-25, *let-7f*

“*” indicates changes in miRNA expression exclusive to breast tumor subtype.

**Table 3:** Predicted miRNAs targeting estrogen receptor genes (ESR1 and ESR2).

MicroRNA Name	Mature Sequence of miRNA	Target Gene		Score Class	References
		*ESR1*	*ESR2*		
hsa-let-7a-5p	6-UGAGGUAGUAGGUUGUAUAGUU-27	Y	Y	High	[180,181]
hsa-let-7b-5p	6-UGAGGUAGUAGGUUGUGUGGUU-27	Y	Y	High	
hsa-let-7c-5p	11-UGAGGUAGUAGGUUGUAUGGUU-32	Y	Y	High	
hsa-let-7d-5p	8-AGAGGUAGUAGGUUGCAUAGUU-29	Y	Y	High	
hsa-let-7e-5p	8-UGAGGUAGGAGGUUGUAUAGUU-29	Y	Y	High	
hsa-let-7f-5p	63-CUAUACAAUCUAUUGCCUUCCC-84	Y	Y	High	
hsa-let-7g-5p	5-UGAGGUAGUAGUUUGUACAGUU-26	Y	Y	High	
hsa-let-7i-5p	6-UGAGGUAGUAGUUUGUGCUGUU-27	Y	Y	High	
hsa-miR-106a-5p	13-AAAAGUGCUUACAGUGCAGGUAG-35	Y	Y	High	
hsa-miR-122-5p	15-UGGAGUGUGACAAUGGUGUUUG-36	Y	Y	High	
hsa-miR-124-3p	14-CGUGUUCACAGCGGACCUUGAU-35	Y	Y	High	
hsa-miR-129-5p	5-CUUUUUGCGGUCUGGGCUUGC-25	Y	Y	High	
hsa-miR-140-5p	23-CAGUGGUUUUACCCUAUGGUAG-44	Y	Y	High	
hsa-miR-145-5p	16-GUCCAGUUUUCCCAGGAAUCCCU-38	Y	Y	High	
hsa-miR-15a-5p	14-UAGCAGCACAUAAUGGUUUGUG-35	Y	Y	High	
hsa-miR-15b-5p	20-UAGCAGCACAUCAUGGUUUACA-41	Y	Y	High	
hsa-miR-16-5p	14-UAGCAGCACGUAAAUAUUGGCG-35	Y	Y	High	
hsa-miR-17-5p	14-CAAAGUGCUUACAGUGCAGGUAG-36	Y	Y	High	
hsa-miR-195-5p	15-UAGCAGCACAGAAAUAUUGGC-35	Y	Y	High	
hsa-miR-196a-5p	7-UAGGUAGUUUCAUGUUGUUGGG-28	Y	Y	High	
hsa-miR-196b-5p	15-UAGGUAGUUUCCUGUUGUUGGG-36	Y	Y	High	
hsa-miR-204-5p	33-UUCCCUUUGUCAUCCUAUGCCU-54	Y	Y	High	
hsa-miR-205-5p	34-UCCUUCAUUCCACCGGAGUCUG-55	Y	Y	High	
hsa-miR-20a-5p	8-UAAAGUGCUUAUAGUGCAGGUAG-30	Y	Y	High	
hsa-miR-20b-5p	6-CAAAGUGCUCAUAGUGCAGGUAG-28	Y	Y	High	
hsa-miR-21-5p	8-UAGCUUAUCAGACUGAUGUUGA-29	Y	Y	High	
hsa-miR-211-5p	26-UUCCCUUUGUCAUCCUUCGCCU-47	Y	Y	High	
hsa-miR-214-3p	30-UGCCUGUCUACACUUGCUGUGC-51	Y	Y	High	
hsa-miR-24-3p	7-UGCCUACUGAGCUGAUAUCAGU-28	Y	Y	High	
hsa-miR-25-3p	14-AGGCGGAGACUUGGGCAAUUG-34	Y	Y	High	
hsa-miR-32-5p	6-UAUUGCACAUUACUAAGUUGCA-27	Y	Y	High	
hsa-miR-330-3p	8-UCUCUGGGCCUGUGUCUUAGGC-39	Y	Y	High	
hsa-miR-338-3p	6-AACAAUAUCCUGGUGCUGAGUG-27	Y	Y	High	
hsa-miR-3619-5p	16-UCAGCAGGCAGGCUGGUGCAGC-37	Y	Y	High	
hsa-miR-363-3p	7-CGGGUGGAUCACGAUGCAAUUU-28	Y	Y	High	
hsa-miR-367-3p	6-ACUGUUGCUAAUAUGCAACUCU-27	Y	Y	High	
hsa-miR-424-5p	11-CAGCAGCAAUUCAUGUUUUGAA-32	Y	Y	High	
hsa-miR-497-5p	24-CAGCAGCACACUGUGGUUUGU-44	Y	Y	High	
hsa-miR-507	56-UUUUGCACCUUUUGGAGUGAA-76	Y	Y	High	
hsa-miR-548l	15-AAAAGUAUUUGCGGGUUUUGUC-36	Y	Y	High	
hsa-miR-573	16-CUGAAGUGAUGUGUAACUGAUCAG-39	Y	Y	High	
hsa-miR-583	16-CAAAGAGGAAGGUCCCAUUAC-36	Y	Y	High	
hsa-miR-590-5p	16-GAGCUUAUUCAUAAAAGUGCAG-37	Y	Y	High	
hsa-miR-7-5p	24-UGGAAGACUAGUGAUUUUGUUGUU-47	Y	Y	High	
hsa-miR-761	7-GCAGCAGGGUGAAACUGACACA-28	Y	Y	High	
hsa-miR-766-3p	29-AGGAGGAAUUGGUGCUGGUCUU-50	Y	Y	High	
hsa-miR-92a-3p	11- AGGUUGGGAUCGGUUGCAAUGCU-33	Y	Y	High	
hsa-miR-93-5p	11-CAAAGUGCUGUUCGUGCAGGUAG-33	Y	Y	High	
hsa-miR-942-5p	13-UCUUCUCUGUUUUGGCCAUGUG-34	Y	Y	High	
hsa-miR-98-5p	22-UGAGGUAGUAAGUUGUAUUGUU-43	Y	Y	High	

* ‘miR’ means microRNA, ‘Y’ represents the gene targeted by a miRNA. High score class indicates that the prediction confidence ranks among the top 5%.

**Table 4:** List of approved or investigational epigenetic drugs for breast cancer treatment.

Drug Category	Drug Name	References
DNA methyltransferase inhibitors	5-Aza-2′-deoxycytidine, 5-Azacytidine, 5-Fluoro-2-Deoxycytidine	[228,229]
Histidine methyltransferase inhibitors	Curcumin	[228]
Histone deacetylase inhibitors	Belinostat, Entinostat, Panobinostat, Suberoylanilide hydroxamic acid (SAHA), Sodium butyrate, Vorinostat, Valproic acid, CUDC-101	[228,229]
Histone methyltransferase inhibitors	EPZ004777, UNC0638	[228,230]

**Table 5:** List of investigational miRNA mimics against breast cancer.

Name	Effect on Breast Cancer Hallmarks	Target Gene/Signaling Pathway	References
miRNA-216a mimics	↓Cell proliferation ↓Cell migration ↑Apoptosis	Wnt/β-catenin signaling	[74]
miRNA-449b-5p mimics	↓ Growth ↓ Invasion	*CREPT*; Wnt/β-catenin/TCF-4 signaling	[92]
miRNA-301b mimics	↓ Cell proliferation ↑ Apoptosis	*CYLD*	[111]
miRNA-1271 mimics	↓ Cell proliferation ↓ Invasion ↓ Migration abilities	*SPIN1*	[134]
miRNA-16 mimics	↑ Apoptosis ↑ Cell-cycle arrest ↓ Invasion and migration	*TGFBR2*, *SMAD2*, *SMAD3*; TGF-β signaling	[264]
miRNA-34a mimics	↑ Apoptosis ↑ Cell-cycle arrest ↓ Invasion and migration	*TGFBR2*, *SMAD2*, *SMAD3*; TGF-β signaling	[264]
miRNA-381 mimics	↓ Cisplatin resistance	*MDR1*	[265]
miRNA-135a mimics	↓ Proliferation ↓ Clongenicity	*ELK1*, *ELK3*	[266]
miRNA-27a mimics	↓ Tamoxifen resistance	*ERα*	[185]
miRNA-98 mimics	↓ Proliferation ↓ Invasion ↓ Migration ↑ Apoptosis	*HMGA2*	[267]

*↑ indicates promotion/induction, ↓ indicates reduction/inhibition.
